# Continuous-flow synthesis of dimethyl fumarate: a powerful small molecule for the treatment of psoriasis and multiple sclerosis†

**DOI:** 10.1039/c9ra09119j

**Published:** 2020-01-16

**Authors:** Marcelo T. Lima, Fernanda G. Finelli, Alline V. B. de Oliveira, Vinicius Kartnaller, João F. Cajaiba, Raquel A. C. Leão, Rodrigo O. M. A. de Souza

**Affiliations:** Biocatalysis and Organic Synthesis Group, Chemistry Institute, Federal University of Rio de Janeiro 21941909 Brazil rodrigosouza@iq.ufrj.br; Instituto de Pesquisas de Produtos Naturais, Federal University of Rio de Janeiro 21941909 Brazil; Núcleo de Desenvolvimento de Processos e Análises Químicas em Tempo Real (NQTR), Chemistry Institute, Federal University of Rio de Janeiro 21941909 Brazil

## Abstract

Dimethyl fumarate (DMF) is a methyl ester of fumaric acid and has recently gained attention due to its use as a pro-drug in different pharmaceutical preparations, besides the low price of the final molecule and no active patents being available for the synthesis of DMF, the prices of multiple sclerosis treatment are still high. In our continuous effort for the development of process intensification strategies towards the synthesis of active pharmaceutical ingredients, here we present our work on a cascade methodology for dimethyl fumarate synthesis in short reaction times and quantitative yields.

## Abstract 2

Dimethyl fumarate (DMF) has recently gained attention due to its use as a pro-drug in different pharmaceutical preparations, here in we present our work on a cascade methodology in short reaction times and quantitative yields.

## Graphical abstract

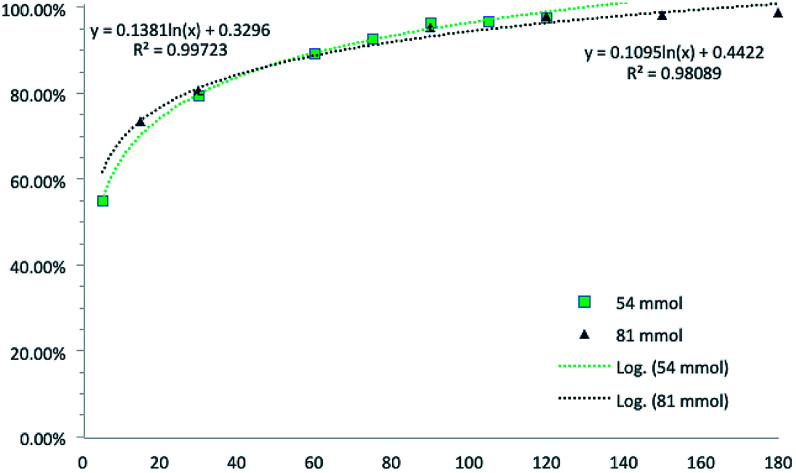

## 1. Introduction

Dimethyl fumarate (DMF) is a methyl ester of fumaric acid and has recently gained attention due to its use as a pro-drug in different pharmaceutical preparations for the treatment of psoriasis and multiple sclerosis. DMF entered the market in 1994 (Germany), under the name Fumaderm®, where it has become the most frequently used first-line systemic psoriasis treatment.^1–3^ Meanwhile it was observed that some patients treated for psoriasis that also have multiple sclerosis had an improvement on their clinical condition.^4^ After 20 years of Fumaderm®, Tecfidera® was launched into the market in 2013, for the treatment of adults with relapsing forms of multiple sclerosis. In 2017, an oral formulation under the brand name Skilarence® was approved in Europe for the treatment of moderate-to-severe plaque psoriasis in adults.^5^ The mechanism of action is not yet well understood but the main activity of DMF and monomethyl fumarate (MMF)^6^ is considered to be immunomodulatory by reducing the expression of micro-RNA-21, which is essential for the production of pathogenic cells in multiple sclerosis and psoriasis.

Besides the low price of the final molecule and no active patents available for the synthesis of DMF, prices of multiple sclerosis treatment are high reaching 75.000 USD per year for nearly 87 g of DMF (860 USD per g) and in 2018, retail sales of Tecfidera® reached 4.296 billion USD.^7^

In our continuous efforts on developing flow chemistry strategies towards the synthesis of active pharmaceutical ingredients, herein we present our results on the continuous-flow synthesis of dimethyl fumarate using two strategies: starting from fumaric acid or maleic anhydride. A prior evaluation and optimization of the batch process was performed, using on-line real-time infrared spectroscopy. This optimization aimed to feed information for the development of a continuous-flow protocol. For a better adjustment from batch to continuous-flow, the DMF crystallization process was also studied by RGB-based image analysis, in order to determine the temperature profile for product crystallization.

## 2. Material and methods

### 2.1. Materials

Reagents were purchased from different sources and used without further purification: fumaric acid >99% and maleic anhydride 99% from Fluka Chemistry, thiourea >99% and sulfuric acid 98% from Sigma-Aldrich and methanol 99% from Tedia Brazil.

^1^H-NMR was recorded on a Bruker Advance 500 MHz spectrometer. Reported chemical shifts (*δ*) are expressed in parts per million (ppm) down field from tetramethylsilane (TMS).

### 2.2. Chromatography analysis

Samples were prepared by stirring 20 μL of reaction crude and 50 μL *N*-methyl-*N*-(trimethylsilyl)trifluoroacetamide (MSTFA) at 60 °C for 30 minutes, followed by addition of 930 μL of methanol. Conversion percentages were analyzed by chromatogram areas using the Shimadzu GC2010 GC-MS – SLB-5MS column 30 meters. Injection temperature 250 °C, injection split ratio 20.0, carrier gas was He, pressure 100.0 kPa, column flow 1.61 mL min^−1^. The oven temperature setting was: 60 °C for 2 min, heated at 7 °C min^−1^ to 160 °C and remained for 1 min. Conversion percentages were analyzed by chromatogram area. Mass ion source temperature 250 °C, interface temperature 300 °C, solvent cut time 3.5 min and scan acquire mode.

### 2.3. Experimental section

#### 2.3.1. Continuous flow synthesis of dimethyl fumarate from fumaric acid

Solution A (fumaric acid in methanol, 0.81 M) and solution B (H_2_SO_4_ in methanol, 0.46 M) were pumped through a T-junction to the reactor zone. After mixing, they proceeded to a 9.8 mL tubular reactor (1.5 mm inner diameter and 3.18 mm outer diameter) heated externally to 55–120 °C, residence time 12–30 min.

#### 2.3.2. Continuous flow synthesis of dimethyl fumarate from maleic anhydride

Solution A (maleic anhydride in methanol, 2.4 M) and solution B (thiourea in methanol, 0.67 M) were pumped through a T-junction to the reactor zone. After mixing, they proceeded to a 16 mL tubular reactor (1.0 mm inner diameter outer diameter) heated externally to 40–105 °C, residence time 7.5–30 min. A solution of H_2_SO_4_ in methanol 0.141 M was subsequently pumped to the reaction outcome. After mixing, they proceed to a 16 mL tubular (1.0 mm inner diameter outer diameter) heated externally to 105 °C, residence time 16 min.

## 3. Results and discussion

**Fig. 1:**
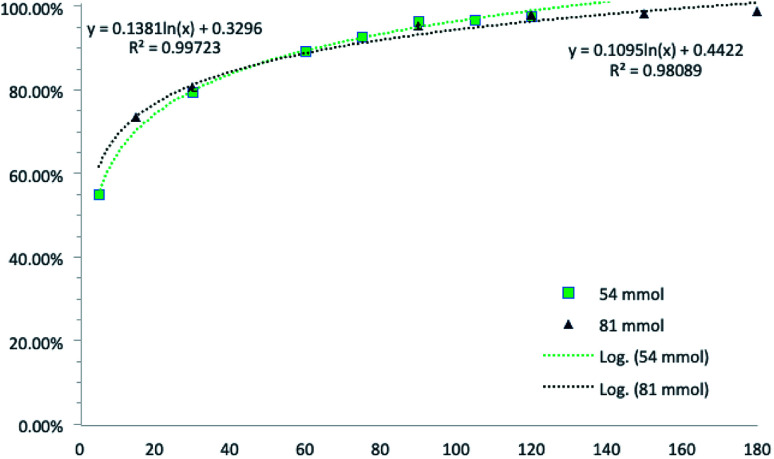
Conversion towards DMF at different concentrations of fumaric acid using H_2_SO_4_ as catalyst at 55 °C. Reaction conditions: 0.22 g (1.9 mmol) or 0.33 g (2.8 mmol) of fumaric acid; 10 mol% of H_2_SO_4_, 3.5 mL of methanol at 55 °C.

**Scheme 1:**
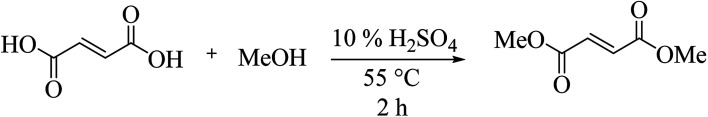
Optimized conditions to synthesis of dimethyl fumarate.

Our studies started with the synthesis of DMF from the batch reaction of fumaric acid in methanol at 65 °C, employing 4 mol% of sulfuric acid as catalyst, according Tachibana's protocol.^8^ With this initial results in hands which reproduces previous works already published over literature, we decided to move forward in order to evaluate the reactions parameters: temperature, catalyst quantity, reaction time and fumaric concentration (Tables S1–S3†). Fig. 1 presents a relationship between the amount of fumaric acid the conversion to dimethyl fumarate over time. As we can see the quantity of fumaric acid has no significantly impact the conversion. Then the best condition is: 810 mM of fumaric acid, 10% of H_2_SO_4_, at 55 °C along 120 min (Scheme 1). After 2 hours of reaction recrystallization lead to DMF (100% purity by GC-MS) was obtained in 83% yield.

**Fig. 2:**
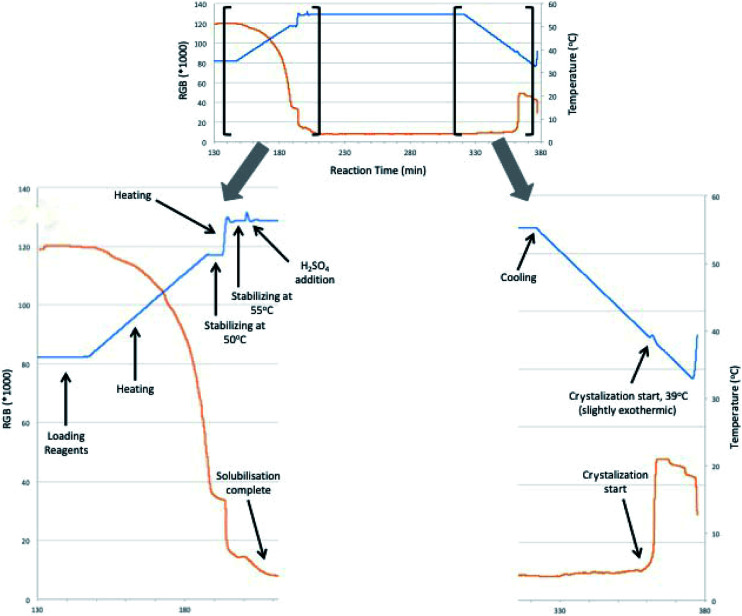
Optimization of dimethyl fumarate synthesis and recrystallization. General reaction conditions: 0.81 mol fumaric acid, 0.081 mol of H_2_SO_4_ and 1000 mL of methanol. The reactor was heated 0.3 °C min^−1^ until 50 °C, after that the rate was increased to 4 °C min^−1^. After reaction complete the reactor was slowly cooled.

With this initial results in hands which reproduces previous works already published over literature, we decided to move forward in order to evaluate the recrystallization process of DMF. Initial conditions took us to very good crystallization yields in the range of 80–85% on a two-step process. These steps consisted in: first cooling the reaction system to 35 °C, followed by filtration of dimethyl fumarate crystals and further crystallization of remaining soluble dimethyl fumarate from the liquid phase (∼10% of solid reminiscent). In order to have more accurate data about the crystallization procedure developed, we have taken advantage of a methodology already extensively used by our group to monitor solid crystallization: the RGB-based image analysis.^9–12^ This technique uses a LED light source and a CCD-camera, with a 90° angle between them, to monitor the crystallization process. When the system is completely homogeneous, before crystallization, the light from the LED source cannot reach the camera, since they are in an orthogonal angle. As soon as particles are formed in the system, they are able to scatter and disperse the light, which then reaches the camera and increase the luminosity of the acquired images. This luminosity is then measured by the RGB (red, green and blue) values, from the RGB color system. The RGB values are directly proportional to the luminosity of the system. Hence, the RGB signal can be used to monitor the crystallization process: when particles are absent from solution, the RGB tends to zero; when particles start to form, the signal increases, proportional to the number of particles. Results extracted from the experiment are shown on Fig. 2.

The crystallization study was performed coupled to a prior reaction, for the formation of DMF in solution. The reaction was carried out using the best reaction conditions optimized previously. After loading the reagents to the reactor, temperature was raised to 50 °C (at a rate of 4 °C min^−1^), stabilized for a few minutes and raised again to 55 °C (1 °C min^−1^). H_2_SO_4_ was added to the reaction medium and complete solubilization of fumaric acid was observed. After reaching the desired reaction time, reactor vessel was cooled (4 °C min^−1^) and the beginning of the crystallization process was observed at 39 °C by the RGB signal (orange line) and calorimetry (blue line). It should be noted that all crystallization processes exhibit an exothermic behavior, that was also observed by measuring the reactional temperature. As crystallization occurred, heat was produced, which increased slightly the medium temperature, before being once again decreased.

**Fig. 3:**
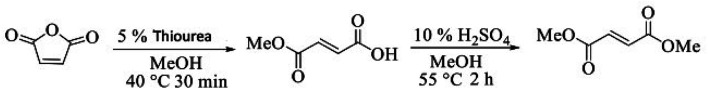
Synthesis of dimethyl fumarate from maleic anhydride.

Our second strategy to synthesize DMF is from a cascade hydrolysis/isomerization of maleic anhydride, followed by acid catalyzed esterification reaction. As the second step was already under our knowledge, we have decided to start optimizing the cascade hydrolysis/methanolysis and isomerization of maleic anhydride following previous protocols obtained from patents. In both cases no more than 30 minutes of reaction was needed to arrive on quantitative conversion of maleic anhydride to fumaric acid or methyl fumarate under thiourea catalysis at 40 °C. The amount of thiourea used, 5, 10 or 15% has no great influence on the conversion towards the desired product (see further details on ESI†). In order to evaluate the cascade reaction we decided to run the first step (methanolysis followed by isomerization) and then add the amount of H_2_SO_4_ needed for the esterification reaction. Starting from a stock solution of maleic anhydride in methanol (2.0 M), after 30 minutes at 40 °C, only methyl fumarate was detected on reaction media. Then, temperature was raised to 55 °C and 15% of H_2_SO_4_ added to the reaction media, after 2 hours, 98.6% of dimethyl fumarate was obtained (Fig. 3).

With these initial results in hands we decided to move forward in order to develop a continuous-flow protocol for both strategies aiming the production of dimethyl fumarate. Starting from fumaric acid approach, adapting the batch protocol to the continuous system was challenging because of systematic blockages due to the high concentration of the reaction media. In order to overcome this issue, we decided to take advantage of the continuous-flow system in order to heat the reaction system above the boiling point of methanol to explore new process windows (Table 1).

**Table 1:** Experimental result of the continuous flow reaction, with residence time of 16 and 12 min at different temperatures^a^

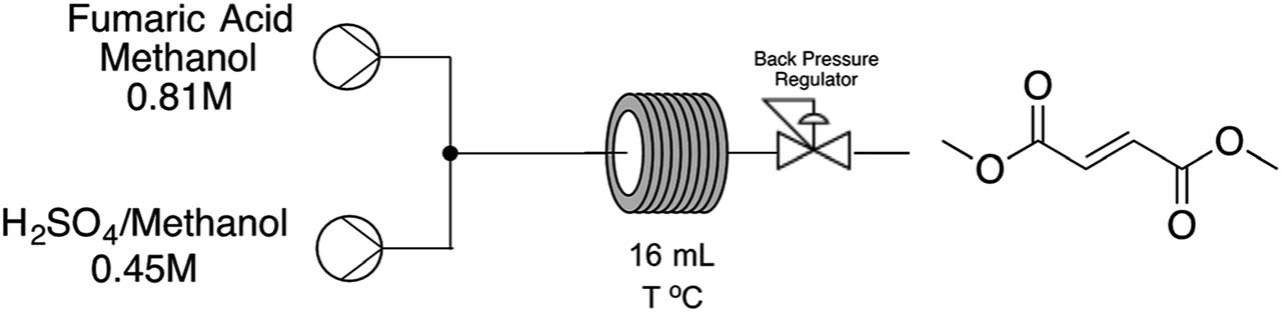			
Entry	Temp (°C)	Res. time (min)	Yield (%)
1	55	16	43
2	65	16	57
3	95	16	73
4	105	16	>99
5	120	12	98

a General reaction conditions: 0.81 M substrate, 0.45 M H_2_SO_4_, 55–120 °C, 16 mL PFA reactor with 1.0 mm internal diameter and 1.6 mm external diameter.

As shown in Table 1, moderate results could be obtained at temperatures ranging from 55 to 95 °C (entries 1–3, Table 1) but a 10 °C increase on reaction temperature could lead to a complete conversion towards the desired product in very short reaction time. A further increase on reaction temperature to 120 °C can lead to a slightly decrease on residence time from 16 to 12 minutes, maintaining the desired yield of the reaction and a space-time-yield of 158.4 g per day. It's important to note that the obtained values shown on Table 1 are related to steady state conditions. In order to verify the reproducibility of the developed process we have decided to increase reactor volume from a 1/16′′ tubing to a 1/8′′. Results obtained show that reaction temperature of 120 °C and residence time of 12 minutes still affords the desired product with similar yields upgrading productivity from 158.4 g per day to 10 200 g per day.

The maleic anhydride approach started by investigating the continuous-flow strategy for both steps separately. First step consisted on methanolysis of maleic anhydride followed by isomerization arriving at methyl fumarate. An initial assessment of reaction temperature and residence time was made and results are presented on Table 2.

**Table 2:** Experimental results of the continuous flow reaction at different residence time and temperatures^a^

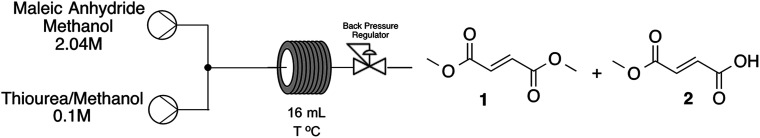				
Entry	Temp. (°C)	Res. time (min)	Product (%)	
			1	2
1	40	30	3.2	92.2
2	105	30	2.7	89.3
3	105	15	1.4	93.5
4	105	7.5	0.5	96.3
5	65	15	0.3	98.8

a General reaction conditions: 2.04 M substrate, 0.1 M thiourea, 40–105 °C, 16 mL PFA reactor with 1.0 mm internal diameter and 1.6 mm external diameter.

Results presented on Table 2 show that excellent conversions can be obtained to the desired products in very short reaction times if temperature is raised above the boiling point of reaction solvent. Under mild conditions excellent results can be obtained at 40 °C and 65 °C with moderate residence times.

**Scheme 2:**
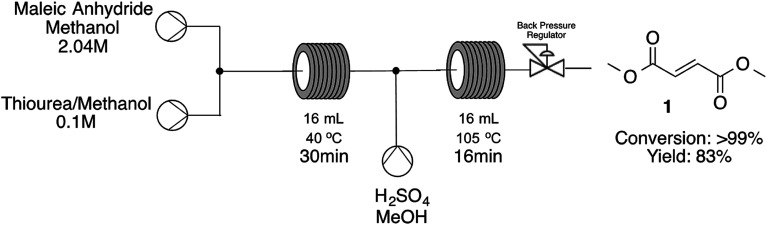
Cascade reaction for dimethyl fumarate synthesis starting from maleic anhydride.

The next step was the opportunity to establish the cascade reaction integrating the methanolysis of maleic anhydride leading to methyl fumarate and consequent esterification to dimethyl fumarate. For the second step we decided to use the expertise already gained during the optimization of fumaric acid esterification and has used such conditions as our first guess (Scheme 2).

As shown on Scheme 2 we have successfully developed a cascade reaction starting from maleic anhydride and arriving at dimethyl fumarate in a single step with quantitative conversions towards the desired product with a STY 4147 g per day. When integrating both steps we have decided to use a mild condition during the methanolysis step in order to ensure a clean and productive procedure. If we have started with high temperatures on the first step, temperature drop by addition of methanolic sulfuric acid solution and connection between the two reaction zones could lead to precipitation of the first intermediate leading to blockages and decreased conversions. The time to get into steady state was 102 minutes and the system was stable without significant variations on conversion for additional 152 minutes.

## 4. Conclusion

In conclusion we have developed a continuous-flow strategy towards the synthesis of dimethyl fumarate through two different approaches: starting from fumaric acid the continuous esterification protocol lead to dimethyl fumarate in quantitative conversion and excellent yield after short residence times (16 minutes) at 105 °C, a great improvement compared to the batch protocol. Starting from maleic anhydride we could also synthesize dimethyl fumarate through a cascade reaction (methanolysis/isomerization/esterification) combining two reaction zones (40 °C and 105 °C) with a total residence time of 46 minutes, leading to the desired product in quantitative conversion (>99%) and excellent yields.

## Conflicts of interest

There are no conflicts to declare.

## Supplementary Material

RA-010-C9RA09119J-s001
