# Supplementary material for: Continuous-flow synthesis of dimethyl fumarate: a powerful small molecule for the treatment of psoriasis and multiple sclerosis
Source: RSC Adv. 2020 Jan 16;10(5):2490–4. doi: 10.1039/c9ra09119j (PMC9048608; doi:10.1039/c9ra09119j)

Electronic Supplementary Material (ESI) for RSC Advances.
This journal is © The Royal Society of Chemistry 2019

Supporting Information for:

Continuous-Flow Synthesis of Dimethyl Fumarate: a Powerful Small Molecule on the Treatment of Psoriasis and Multiple Sclerosis

Marcelo T. Lima [a,b], Fernanda G. Finelli[b], Alline V. B. de Oliveira [c], Vinicius Kartnaller[c],

João F. Cajaiba[c], Raquel A. C. Leão [a], Rodrigo O. M. A. de Souza [a]

a – Biocatalysis and Organic Synthesis Group, Chemistry Institute, Federal University of Rio de Janeiro, 21941909, Brazil;

b – Instituto de Pesquisa em Produtos Naturais, Federal University of Rio de Janeiro, 21941909, Brazil;

c- Núcleo de Desenvolvimento de Processos e Análises Químicas em Tempo Real (NQTR), Chemistry Institute, Federal University of Rio de Janeiro, 21941909, Brazil;

Keywords: continuous-flow, dimethyl fumarate, cascade reactions, api synthesis;

*souzarod21@gmail.com

CONTENTS ESPECTRAL DATA FOR COMPOUNDS:

	**Experimental Section** ………………………………………	S1
1	EXPERIMENTAL PROCEDURE ……………………………	S2
**1.1**	**Batch synthesis of dimethyl fumarate from fumaric acid:** …………………	S2
**1.2**	**Batch synthesis of dimethyl fumarate from maleic anhydride:** ……………	S2
2.	STUDY OF OPTIMIZATION IN BATCH …………………………	S3
**2.1**	**From the esterification of fumaric acid** …………………………………	S3-S4
**2.2**	**Study of optimization from the esterification of maleic anhydride in batch** …………………………………………	S4-S5
3.	CHOMATOGRAPHIC ANALYSIS …………………………………	S5
**3.1**	**Esterification of fumaric acid** ………………………………………	S5
**3.1**	**Esterification of maleic anhydride** …………………………………	S6
4.	SPECTRAL DATA …………………………………………………	S6
**4.1**	**^{1}H-NMR spectra:** Dimethyl fumarate …………………………	S6

5. Pictures from the RGB system used for crystallization S7

1. EXPERIMENTAL SECTION

1.1 Batch synthesis of dimethyl fumarate from fumaric acid:

To a 125mL flask was added 5.0423 g (43.4 mmol) and 80 mL of methanol, and heated to 55 ° C. Then 0.587 mL of H_2SO_4 was added, the mixture was stirred for 120 min. After this time, reaction was cooled to room temperature and allowed to crystallize under stirring for 8 h. The formed solid was filtered off and recrystallized with a 20 % methanol: water solution, filtered and dried at room temperature to give a crystalline solid in 83 % yield.

1.2 Batch synthesis of dimethyl fumarate from maleic anhydride:

To a 50 mL flask was added 10.0469 g (102.5 mmol) and 50 mL methanol, and heated to 40 ° C. Then 0.3130 g (4.0 mmol) of maleic anhydride was added under constant stirring. The conditions were maintained for 30 min. Following 0.815 mL H_2SO_4 was added, and the temperature was raised to 55 °C, the mixture was stirred for 120 min. After that, was cooled at room temperature, crystallization go on under stirring for 8 h. The solid formed was filtered and recrystallized with a 20 % methanol : water solution, filtered and dried at room temperature to give a 65 % yield of crystalline solid.

2. STUDY OF OPTIMIZATION

2.1 From the esterification of fumaric acid in batch

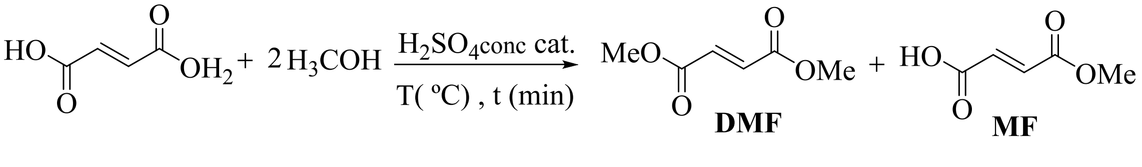

Below is presented the results of parameters optimization (temperature, amount of catalyst and substrate) from the esterification of fumaric acid:

Table S1: Results from the temperature study.

T / t (min.)	55 °C		45 °C		35 °C	
	**MF (%)**	**DMF (%)**	**MF (%)**	**DMF (%)**	**MF (%)**	**DMF (%)**
5	62.4	3.6	35.3	2.4	0	0
10	54.4	5.9	44.2	5.5	0	0
15	47.0	5.7	52.0	9.0	2.46	0
20	52.7	8.0	53.4	9.6	7.22	0
30	55.0	11.6	59.8	17.1	4.45	0

Experimental Conditions: Fumaric acid 0.81 M, 0.081 M of $H_2SO_{4conc.}$ (25 mol%) in methanol, 30 min at 55 °C. *Analyze Conditions*: conversion calculated at GC-MS, oven programming, 60 °C for 1 min, heated for 7 °C/min until 170 °C and maintained 1 min.

Table S2: Results from the catalyst study.

Cat. conc. / t (min.)	5 % mol		7 % mol		10 % mol	
	**MF (%)**	**DMF (%)**	**MF (%)**	**DMF (%)**	**MF (%)**	**DMF (%)**
5	34.9	2.2	33.8	2.5	28.8	3.1
10	33.6	6.5	40.4	4.7	37.2	4.3
15	41.5	5.5	46.2	8.1	44.0	7.2
20	47.1	7.1	50.6	9.6	47.7	10.3
30	51.1	10.3	59.1	15.0	56.3	15.1

Experimental Conditions: Fumaric acid 0.81 M, $H_2SO_{4conc.}$ as catalyst in methanol, 30 min at 55 °C. *Analyze Conditions*: conversion calculated at GC-MS, oven programming, 60 °C for 1 min, heated for 7 °C/min until 170 °C and maintained 1 min.

Table S3: Results from the fumaric acid concentration study.

Fumaric Ac. Conc. / t (min.)	54 mmol		81 mmol	
	**MF (%)**	**DMF (%)**	**MF (%)**	**DMF (%)**
15	39.0	60.3	27.7	72.4
30	22.3	77.7	21.5	78.5
60	11.8	88.1	13.2	86.8
90	3.6	96.4	4.6	95.4

120	2.8	97.2	2.0	98.0
150	0	100	0	100
180	0	100	0	100

Experimental Conditions: Fumaric acid, 10 mol % of $H_2SO_{4conc.}$ as catalyst in methanol, 30 min at 55 °C. *Analyze Conditions*: conversion calculated at GC-MS, oven programming, 60°C for 1 min, heated for 7 °C/min until 170 °C and maintained 1 min.

2.2 Study of optimization from the esterification of maleic anhydride in batch

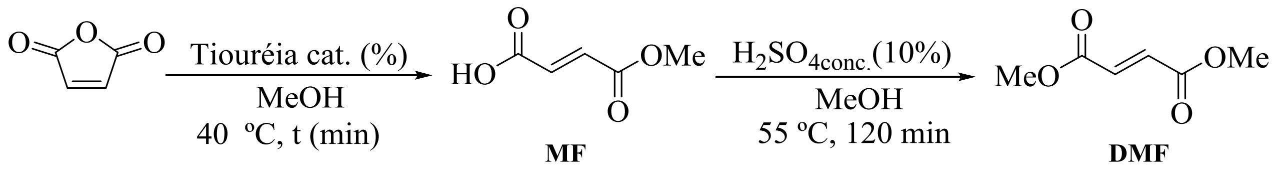

Table S4: Results from the time study.

Time (min)	DMF (%)	MF (%)	Anhydride(%)
60	1.3	98.0	0.7
120	1.6	96.5	1.9
180	2.0	94.6	3.4
240	2.2	95.6	2.1
300	2.5	95.4	2.1
360	2.9	94.9	2.1

Experimental Conditions: Anhydride 2.0 M, 0.1 M of thiourea (5 mol%) in methanol, at 40 °C. *Analyze Conditions*: conversion calculated at GC-MS, oven programming, 60 °C for 1 min, heated for 15 °C/min until 118 °C, heated for 20 °C/min until 150 °C, heated for 15 °C/min until 180 °C and maintained 1 min.

Table S5: Results from the catalyst study with time variation.

Cat. / Time (min)	5%			10%			15%		
	**DMF (%)**	**MF (%)**	**Anhydride (%)**	**DMF (%)**	**MF (%)**	**Anhydride (%)**	**DMF (%)**	**MF (%)**	**Anhydride (%)**
30	0.6	98.3	1.1	0.4	98.4	1.2	0.4	97.6	2.0
60	0.6	98.3	1.1	0.4	98.4	1.3	0.4	97.7	1.9
75	0.7	98.2	1.1	0.4	97.8	1.9	0.4	97.6	2.0
90	0.7	98.1	1.2	0.4	97.6	2.0	0.4	97.7	1.9
120	0.8	98.6	0.7	0.4	97.8	1.7	0.4	97.4	2.1

Experimental Conditions: Anhydride 2.0 M, thiourea as catalyst in methanol, at 40 °C. *Analyze Conditions*: conversion calculated at GC-MS, oven programming, 60 °C for 1 min, heated for 15 °C/min until 118 °C, heated for 20 °C/min until 150 °C, heated for 15 °C/min until 180 °C and maintained 1 min.

Table S6: Results from the cascade reaction.

Time (min)	DMF (%)	MF (%)	Anhydride (%)
**30**	0.2	98.4	1.4
**90**	95.0	3.7	0.0
**150**	98.6	0.9	0.0

Experimental Conditions: Anhydride 2.0 M, 0.1 M of thiourea (5 mol%) in methanol, at 40 °C, after 30 min the H_2SO_4 was added and the temperature was raised to 55 °C. *Analyze Conditions*: conversion calculated at GC-MS; oven programming, 60 °C for 2 min, heated for 15 °C/min until 80 °C, heated for 10 °C/min until 135 °C, heated for 20 °C/min until 140 °C and maintained 1 min, heated for 20 °C/min until 180 °C and maintained 1 min.

3. CHOMATOGRAPHIC ANALYSIS

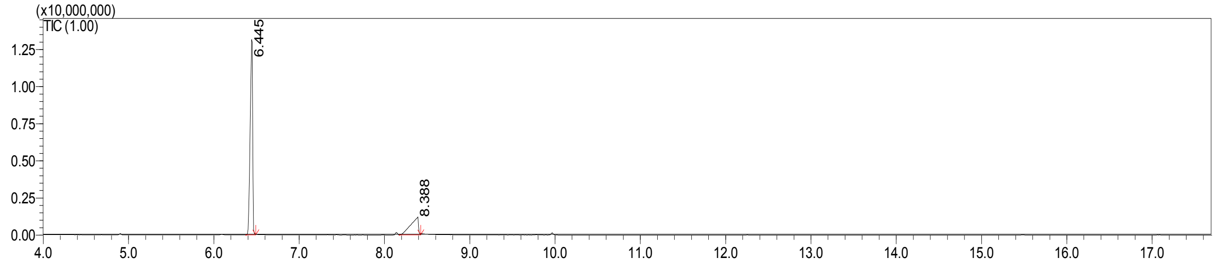

Figure S1: Chromatographic profile obtained by GC-MS after the optimized batch conditions. Retention time: 6.4 min –Dimethyl fumarate, 8.3 min – Methyl fumarate; *Experimental Conditions*: Fumaric acid 0.81 M, 10 mol% of $H_2SO_{4conc.}$ in methanol, 2 h at 55 °C. *Analyze Conditions*: oven programming, 60 °C for 1 min, heated for 7 °C/min until 170 °C and maintained 1 min.

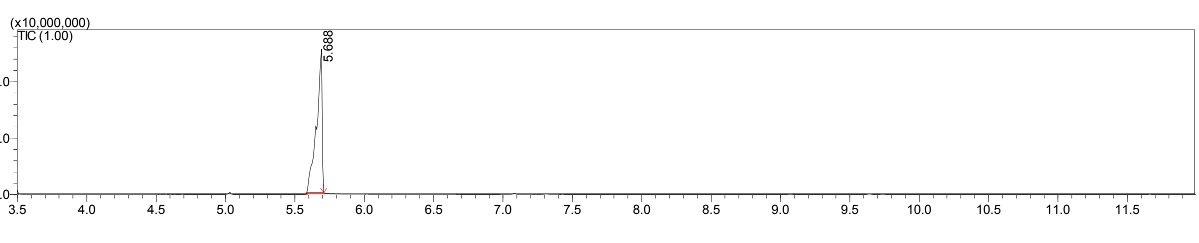

Figure S2: Chromatographic profile obtained by GC-MS after the optimized batch conditions for anhydride. Retention time: 6.4 min –Dimethyl fumarate; *Experimental Conditions*: Anhydride 2.0 M, 0.1 M of thiourea (5 mol%) thiourea in methanol, at 40 °C, after 30 min the H_2SO_4 was added and the temperature was raised to 55°C. *Analyze Conditions*: conversion calculated at GC-MS; oven programming, 60 °C for 2 min, heated for 15 °C/min until 80 °C,

heated for 10 °C/min until 135 °C, heated for 20 °C/min until 140 °C and maintained 1 min, heated for 20 °C/min until 180 °C and maintained 1 min.

4. ESPECTRAL DATA

4.1 ^{1}H-NMR spectra: Dimethyl fumarate (DMF).

^{1}H NMR (500 MHz, $CDCl_3$) δ 6.82 (s, 1H), 3.77 (s, 3H).

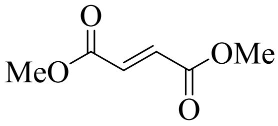

DMF

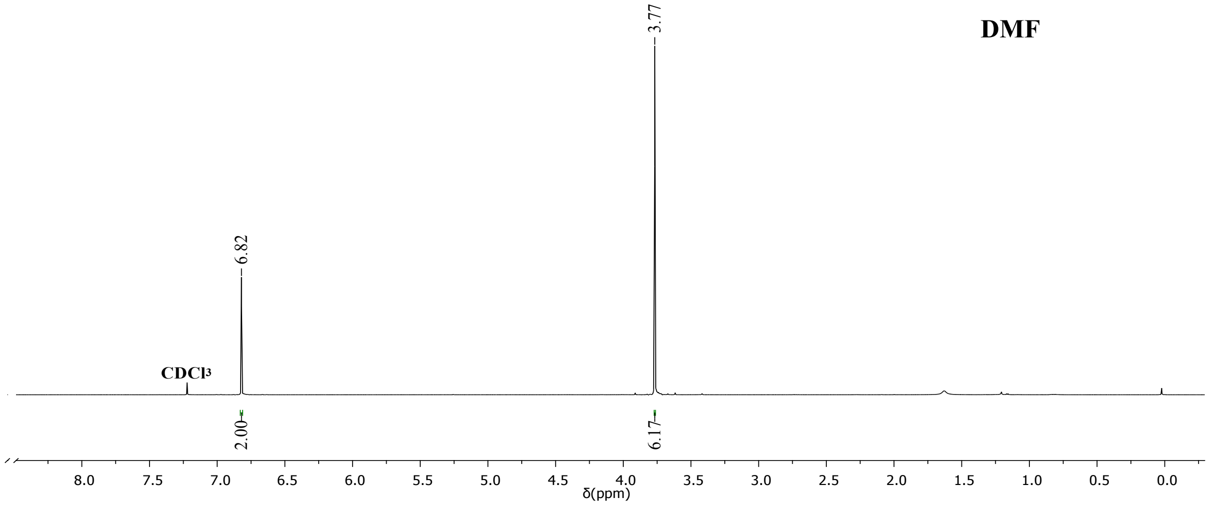

Figure S3: Recrystallized Dimethyl Fumarate (DMF).

5. Pictures from the RGB system used for crystallization:

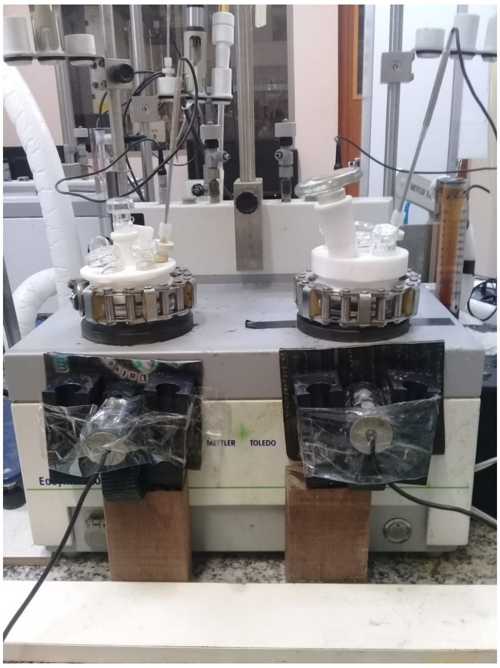
METTLER TOLEDO

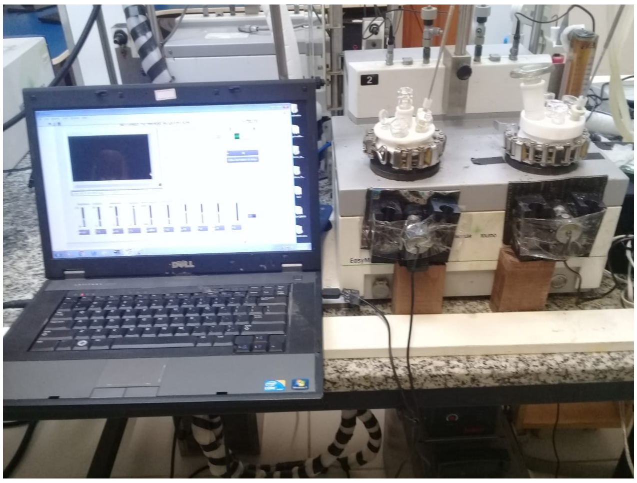

Supplement: RA-010-C9RA09119J-s001 [file RA-010-C9RA09119J-s001.pdf]
